# A stacking-based artificial intelligence framework for an effective detection and localization of colon polyps

**DOI:** 10.1038/s41598-022-21574-w

**Published:** 2022-10-21

**Authors:** Carina Albuquerque, Roberto Henriques, Mauro Castelli

**Affiliations:** NOVA Information Management School (NOVA IMS), Universidade Nova de Lisboa, Campus de Campolide, 1070-312 Lisbon, Portugal

**Keywords:** Biomedical engineering, Cancer imaging, Machine learning

## Abstract

Polyp detection through colonoscopy is a widely used method to prevent colorectal cancer. The automation of this process aided by artificial intelligence allows faster and improved detection of polyps that can be missed during a standard colonoscopy. In this work, we propose to implement various object detection algorithms for polyp detection. To improve the mean average precision (mAP) of the detection, we combine the baseline models through a stacking approach. The experiments demonstrate the potential of this new methodology, which can reduce the workload for oncologists and increase the precision of the localization of polyps. Our proposal achieves a mAP of 0.86, translated into an improvement of 34.9% compared to the best baseline model and 28.8% with respect to the weighted boxes fusion ensemble technique.

## Introduction

In the United States, colorectal cancer (CRC) stands as the third leading cause of cancer-related deaths and it is expected to cause more than 50.000 fatalities by 2022^1^. Additionally, recent studies show that CRC incidence in adults younger than 50 years old has nearly doubled since the early 1990s^2^. Colonoscopy is considered the most effective procedure to detect colon polyps and cancer^3^ and is of paramount importance for effective prevention and reduced risk of death from CRC. Evidence suggests that having a colonoscopy was associated with a decrease of 67% in the risk of death from CRC^4^ and a 70% reduction in the incidence of late-stage CRCs^5^. However, research has shown that in patients undergoing colonoscopy, 25% of polyps are missed^6^. Reasons behind the oversight include overloaded healthcare systems, the presence of flat and small-sized polyps, or workers’ lack of experience^7–9^.

With the rise of artificial intelligence, significant technological advances have occurred in the medical and healthcare field^10^. Deep learning (DL) is widely used as a computer vision tool to classify and detect lesions and many diseases by efficiently addressing the unique challenges of medical data^11^.

In polyp detection, evidence shows that using convolutional neural networks (CNNs) to detect polyps automatically under colonoscopy can improve the detection rate. Qadir et al.^12^ proposed a single-shot feed-forward fully convolutional neural network to develop a real-time polyp detection model using two-dimensional Gaussian masks. Li et al.^13^ used an adaptive training sample to select high-quality training samples to improve generalizability on the accurate segmentation of polyps. Taş et al.^14^ proposed implementing Faster R-CNN with a preprocessing approach based on a super-resolution method to improve the model’s performance in detecting colon polyps. Tang et al.^15^ also used Faster R-CNN with transfer learning to improve polyp detection. The YOLO algorithm has also been proposed to improve the efficiency of polyp detection. Guo et al.^16^ proposed an automatic polyp detection framework based on Yolov3 and active learning to reduce the rate of false positive polyp detection. Pacal et al.^17^ considered Yolov4 for real-time polyp detection, and Wan et al.^18^ used YOLOv5 for the same purpose. Jha et al.^19^ applied EfficientDet, RetinaNet, Faster R-CNN, and YOLOv4 to compare their performance on polyp segmentation. Wu et al.^20^ compared UNet, Faster R-CNN, R-FCN, RetinaNet, Yolov3, FCOS, and PraNet and presented a spatial–temporal feature transformation to detect and localize polyps in endoscopy videos automatically.

Ensemble techniques were also considered to improve the polyp detection task. Sharma et al.^21^ applied a voting ensemble technique combining the results of ResNet101, GoogLeNet, and Xception for polyp classification. Younas et al.^22^ proposed a similar approach by implementing a weighted ensemble of GoogleNet and ResNet50, among others, to improve the accuracy of the polyp class identification. In segmentation, DivergentNets^23^ combines five models, and masks are averaged to make the final segmentation mask. In object detection, Hong et al.^24^ and Polat et al.^25^ used weighted boxes fusion methods as an ensemble technique to combine predictions from different models.

The purpose of our study was to analyse the efficacy of implementing a stacking approach to combine the predictions of distinct object detection techniques with the goal of improving the precision in polyp detection.

## Methods

### Baseline models

In this study, we approach the polyp detection problem using five well-known object detection algorithms proposed in the literature.

Faster R-CNN, defined by Ren et al.^26^, is a two-stage object detection model, where in the first module, regions of interest are proposed, and in the second stage, Fast R-CNN^27^ is applied to detect the final boxes and classify them.

Fully Convolutional One-Stage Object Detection (FCOS) is an anchor-box-free single-stage object detection model proposed by Tian et al.^28^ By eliminating the predefined set of anchor boxes and all related hyperparameters, FCOS avoids computation related to this aspect, with the advantage of being a more straightforward and solid alternative to other object detection algorithms.

RetinaNet^29^ is a one-stage framework that uses focal loss to prevent the high number of negative detections from overwhelming the detector during training.

EfficientDet^30^ is a single-shot detector that uses EfficientNets^31^ as the backbone network along with weighted bidirectional feature networks for feature fusion.

Ultralytics^32^ proposed YOLOv5 as a recent update to the YOLO family of models. YOLO algorithms are characterized by being the first object detection model that combined bounding box prediction and object classification into a single end-to-end differentiable network.

Although one-stage detectors have high inference speed, two stage-detectors are known for their high localization capability and recognition accuracy.

### Ensemble techniques

To compare our method against other ensemble algorithms, we evaluate the performance of four distinct algorithms, considering six variants in total.

In Non-Maximum Suppression (NMS)^33^, all detection boxes are sorted according to their confidence scores, and the detection box D with the maximum score is selected, while the remaining boxes that overlap D more than a predefined threshold are suppressed. These steps are recursively applied to the remaining boxes.

In Soft-NMS^34^, the authors propose a simple change to NMS to surpass the NMS limitation where detection proposals with high Intersection over Union (IoU) and high confidence can be removed. The algorithm decays the detection scores of all the detection boxes as a continuous function of their overlap with D. Two versions of Soft-NMS are tested in this study. In the first version a Gaussian distribution is implemented to modify the detection scores, whereas in the second, a linear function is used.

In Non-Maximum Weighted (NMW)^35^, all detection boxes are considered, and a weighted box is created using IoU values. In this algorithm, the confidence scores are not changed, and the IoU value is used to weight the boxes. Furthermore, NMW does not consider the number of models used in the ensemble.

In Weighted Boxes Fusion (WBF)^36^, similar to NMW, all detection boxes are considered to create a weighted box. However, in WBF, the confidence value is changed using an average value of all the boxes used in each fusion. The coordinate of the fused box is a weighted sum of coordinates of each box where weights are the confidence for boxes. In this case, the boxes with more significant scores will have more influence in defining the coordinates of the fused box than boxes with lower scores would have. A second version of this approach is applied, WBF maximum, where confidence in weighted boxes is calculated using the maximum value instead of using an average value.

### Multistage algorithms

Cascade R-CNN^37^ is a multistage object detection algorithm, considered an extension of R-CNN, where stages are trained sequentially, using the output of one stage to train the next one. By adjusting the bounding boxes at each stage, this approach tries to optimize the IoU values, which sequentially allows the algorithm to be more selective against close false positives for training the next stage.

### Our proposal: StackBox

In this work, we propose a novel ensemble technique to combine the predictions of different models into a final improved prediction. In the stacking approach, we combine multiple algorithms via meta-learning. This procedure involves two or more base models, often referred to as level-0 models or base learners, and a meta-model (which is also called a level-1 model) that combines the predictions of the level-0 models. In stacking, base learners fit on the training data, and those predictions are combined at the end; the resulting combination is then added as input features in the meta-model.

**Figure 1:**
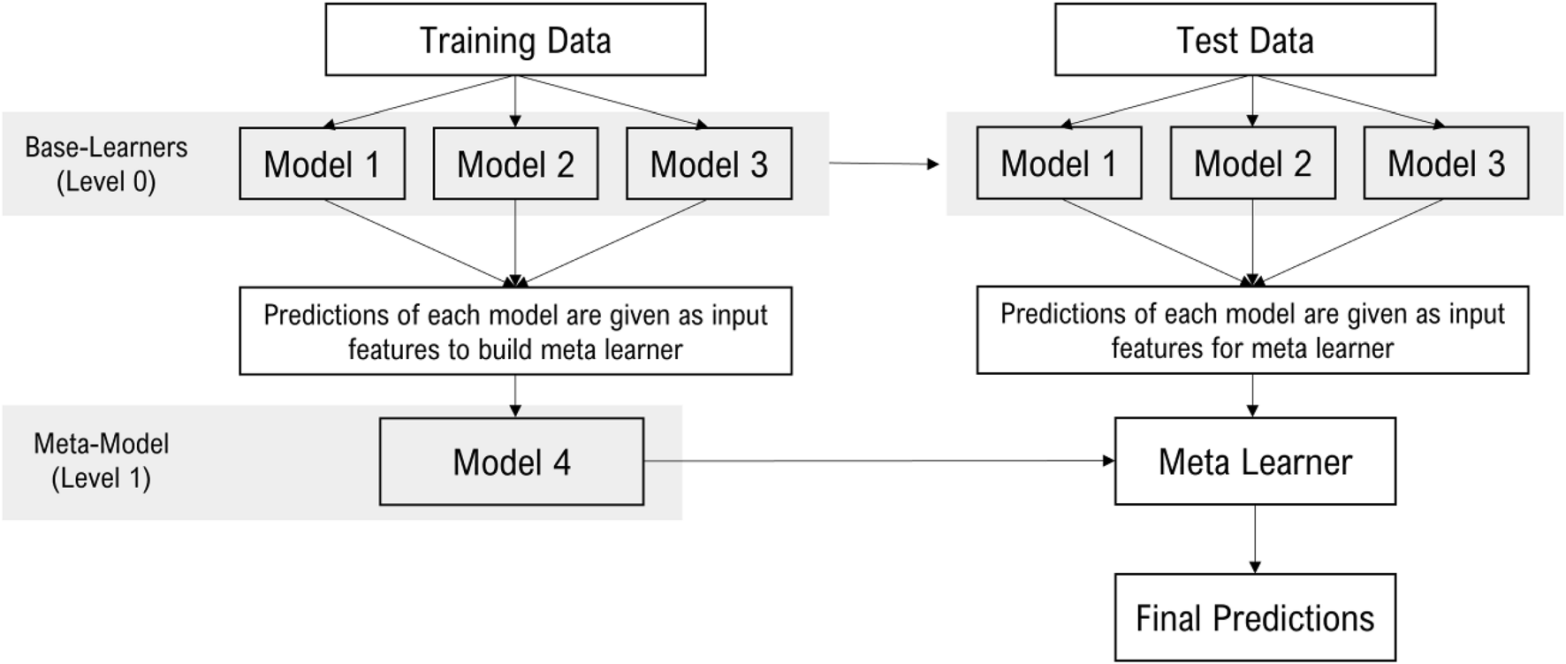
Illustration of StackBox framework. The framework builds distinct base learners (using the training data), and from these models predicts the bounding boxes around the detected objects. Using these predictions as input and using the ground-truth as output, a meta-model combines the base learners’ output, building a new model with improved performance. The base learners built previously are subsequently applied to the test data set to detect polyps on unseen data. Finally, these detections are used as input features for the meta-learner built on the training data to obtain the final predictions on the test data set.

StackBox is a stacking technique that uses a machine-learning model to learn how best to combine the predictions from contributing base learners. Based on the training data set's predictions, base learners (level 0) are combined and are trained using a meta-model (level 1). This stacking technique will combine the capabilities of different base learners, which in this case are traditional object detection algorithms, and the meta-model, a traditional machine learning regressor, trained using the predictions of the base learners on training data, which can be subsequently used to predict new coordinates on the test data, using as input the predictions in the test set, as seen in Fig. 1. When applying StackBox, a different treatment is used in training and test data.

In training data, we assume that the target of the meta-model is the ground truth bounding box, and the input is the base models’ predictions that have the highest IoU associated with the ground truth. In a ground truth where no prediction is available (i.e., where no predicted box is found in any of the models), applying the meta-learner will not be considered. In case the number of predictions available for a specific ground truth is lower than the number of base models used, the missing predictions will be replaced by the values of the predicted box with the highest IoU, independently of the model. In this way, each ground truth will be associated with different predicted boxes, in the same number as the base learners.

**Figure 2:**
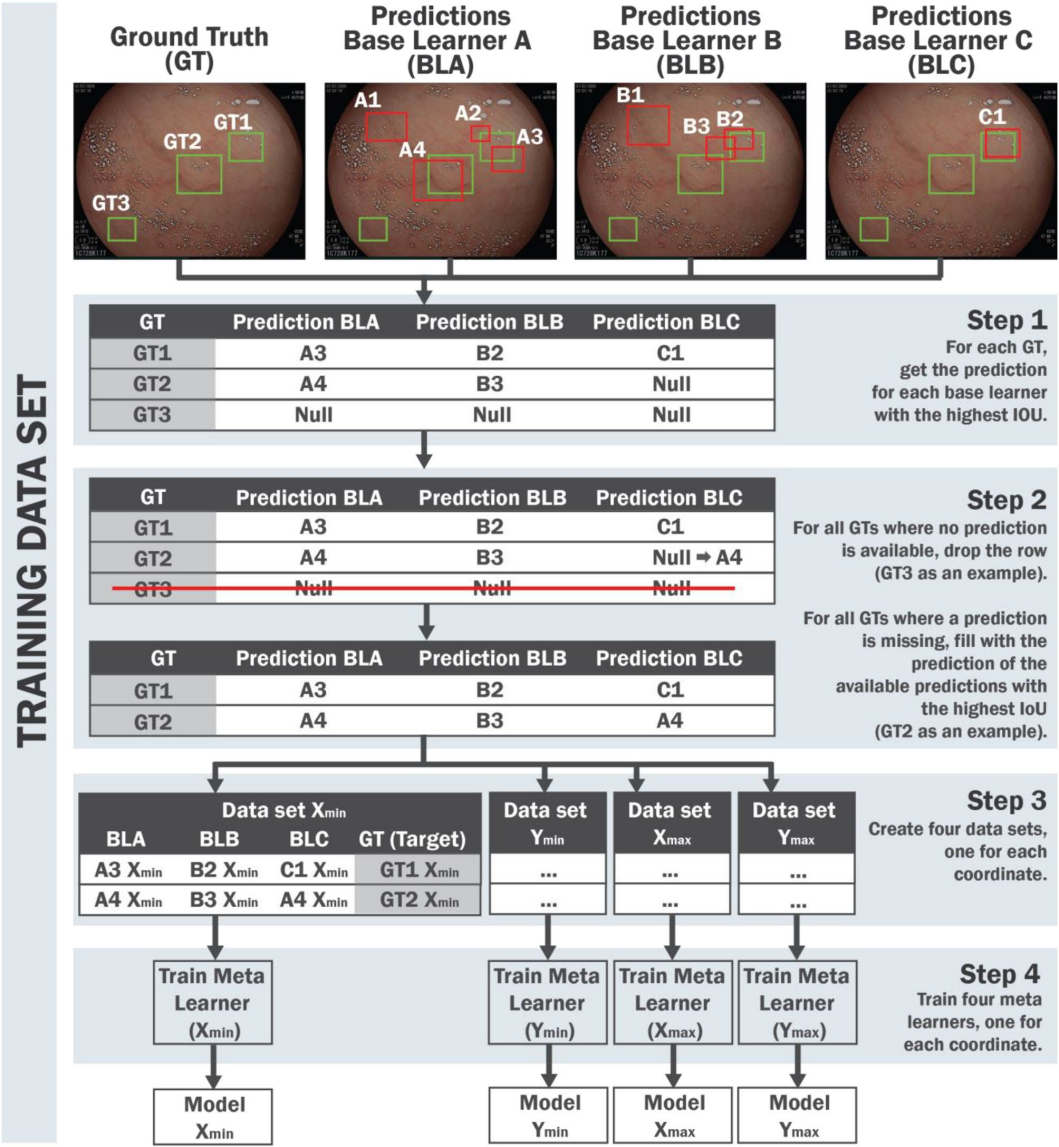
StackBox over the training data. In Step 1, for each ground-truth, we find the prediction of each base learner with the highest IoU. In Step 2, the ground truths without associated predictions are removed; when the number of predictions is lower than the number of base learner models used, the null values are filled with the prediction where the IoU with the ground-truth is higher. In Step 3, four data sets are created according to the coordinates available for each bounding box. The predictors include the corresponding coordinate of each base learner, and the target is the analogous coordinate of the ground truth. In Step 4, a meta-learner is applied to each data set, and the final models are saved for subsequently application to test data. The ground truth is represented by the green bounding boxes and the predictions by the red bounding boxes.

In object detection, each object of interest is outlined by a bounding box, determined by the x and y coordinates. In this way, each predicted box would be represented by four coordinates, namely x_min_, y_min_, x_max_ and y_max_, where min and max stands for minimum and maximum value. Thus, as can be seen in Step 2 of Fig. 2, each ground-truth is associated with a set of coordinates (and the cardinality of this set corresponds to the number of the base learners). Subsequently, each coordinate (x_min_, y_min_, x_max_, and y_max_) will be split, and a meta-model will be applied to each of them. More specifically, each coordinate individually will be considered to apply a meta-learner. As an example, for x_min_, a new data set is built where the number of rows is the same as the number of objects of interest, and the input features are the predictions of the coordinate x_min_ obtained by each base learner, while the output is the x_min_ of the ground truth. Figure 2 shows all the steps of the proposed StackBox technique when processing the training data.

**Figure 3:**
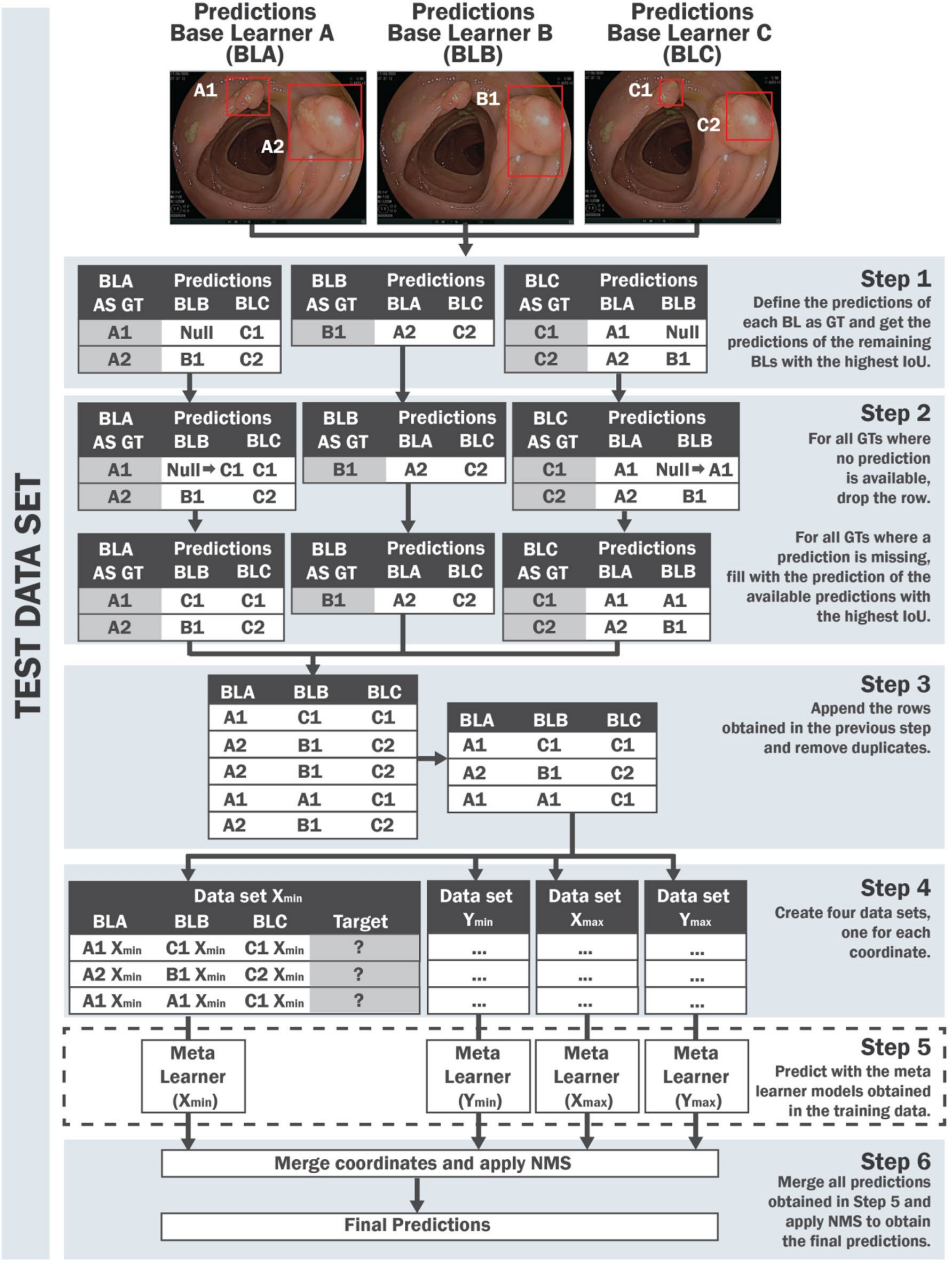
StackBox over the test data. In Step 1, the predictions of each base learner are considered as the ground truth, one at a time, and the predictions returned by the remaining models that have the highest IoU with the ground truth are chosen. In Step 2, similar to the method used in the training data, the ground truths without associated predictions are removed. When the number of predictions is lower than the number of base learner models used minus one, the null values are filled with the prediction where the IoU with the ground truth is higher. In Step 3, all matches obtained in the previous step are concatenated, and the duplicates are removed. In Step 4, four data sets are created according to the coordinates available for each bounding box. The predictors include the corresponding coordinate of each base learner, and the target is predicted using the meta learner models obtained in training data (Step 5). In Step 6, the predictions are combined, and NMS (with a threshold of 0.5) is applied to remove redundant boxes. The red boxes represent the predictions of the base learner models in the test data.

In the test set, we need to define the boxes that will be the input for the meta-learner acquired on training data. At this point, we consider each model’s prediction in the test data as the ground truth. For each prediction in a first model, we find the boxes from the remaining models with the highest IoU and repeat the process for them. This process will lead to several duplicated inputs. All duplicated inputs are removed, and finally we apply the meta-learner obtained in training data to predict the new boxes. Afterward, we apply a NMS strategy to all predictions to remove boxes with an IoU overlap higher than 0.5, keeping the one with the highest confidence. Figure 3 shows all the steps of the proposed StackBox technique for the analysis of the test set. The source code is publicly available at https://github.com/calbuquerque-novaims/StackBox.

**Figure 4:**
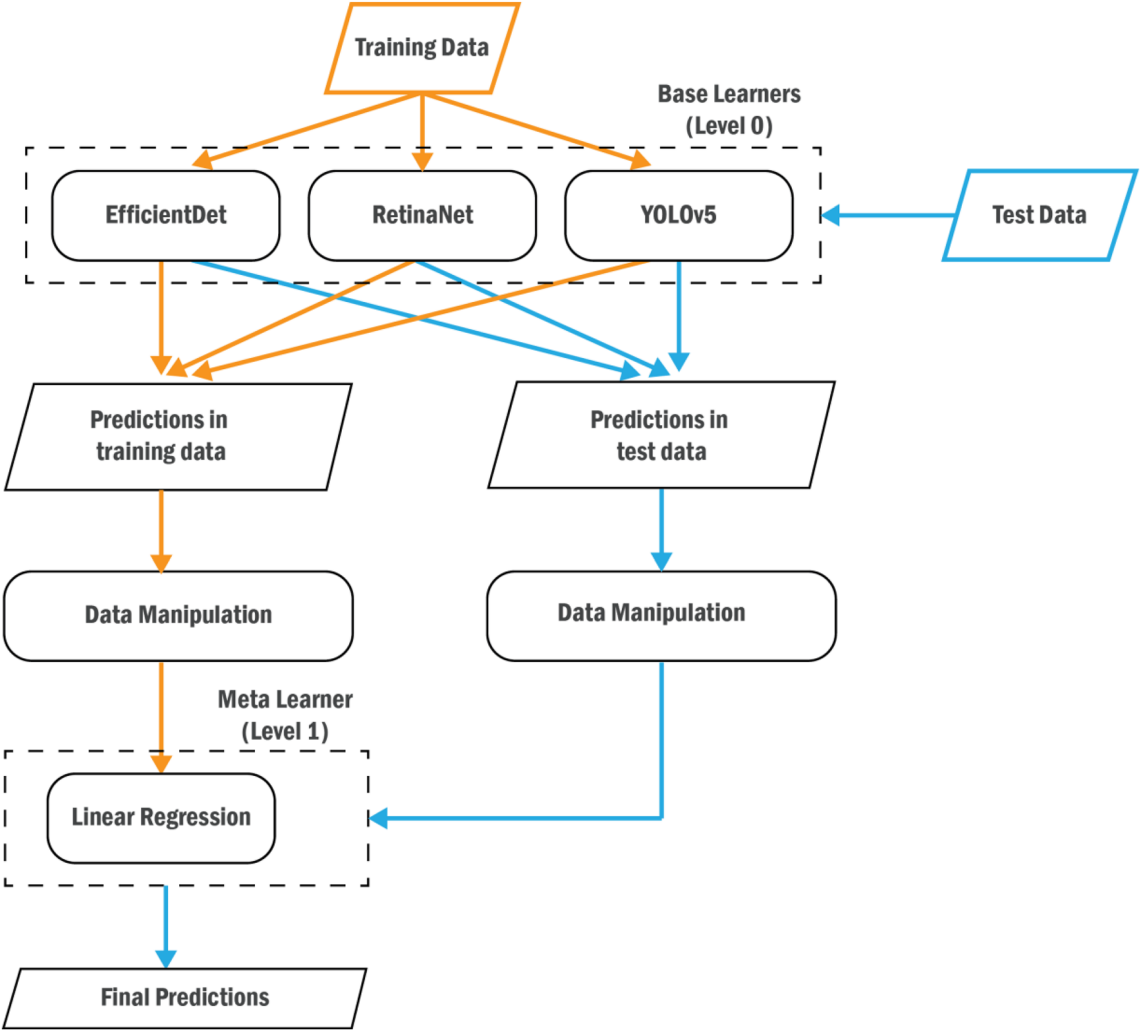
StackBox general overview. The orange flow represents the training stage, and the blue flow represents the testing stage. The data manipulation process in the training stage includes Steps 1 to 3, detailed in Fig. 2. The data manipulation process in the testing phase comprises the Steps 1 to 4, described in Fig. 3.

Figure 4 shows an overview of the StackBox workflow, where the considered meta-learner is the Linear Regression.

We tested different machine learning models as meta-learner. Results show the performance of our stacking technique by applying Linear Regression (LR), Adaboost, Random Forest (RF), GradientBoosting (GB), and XGBoost.

To validate the effectiveness of our proposal, we perform three experiments:

- A comparison with baseline models, where we compare our stacking technique with five widely used object detection models: Faster R-CNN, FCOS, RetinaNet, EfficientDet, and YOLOv5.
- A comparison of our stacking approach with some available ensemble techniques: NMS, Soft-NMS NMW, and WBF.
- A comparison with a multistage approach, Cascade R-CNN.

In all experiments, standard metrics for object detection^38^ are employed for performance measurement, namely AP@[.5:.05:.95], AP@.50, AP@.75, AP_M_, AP_L_, AR_1_, AR_10_, AR_M_, AR_L_, and mAP (IOU = .50).

### Polyp data set

BKAI-IGH Neopolyp-Small^39,40^, a data set of 1000 annotated endoscopic images provided publicly by BK.AI, Hanoi University of Science and Technology incorporation with the Institute of Gastroenterology and Hepatology (IGH), is curated to train and benchmark the proposed approach. The images were collected in IGH, and annotations were added and verified by two experienced endoscopists in IGH.

Originally developed as a segmentation problem, annotations in the data set were converted to a detection problem, where a bounding box identifies each polyp. The data set is randomly split into a training set of 800 images and a test set of 200 images. A fivefold cross-validation approach was used to measure the performance of each of the base models, and the ensembles applied, with no overlapping, and average scores were calculated. The original size of the images is not constant and ranges from 959 × 1280 pixels to 1024 × 1280 pixels. On training, all images were converted to 640 × 640 pixels.

### Experimental setup

All experiments were conducted using models provided by IceVision, a framework for object detection and deep learning that offers an end-to-end workflow with different models from TorchVision, Open MMLab’s MMDetection, and Ultralytic’s YOLOv5, among others. Each base learner model was trained during 50 epochs, and we applied transfer learning using a previously trained model on the Microsoft COCO^41^ data set. As a backbone, we used ResNet101 in RetinaNet, Faster R-CNN and FCOS, D1 in EfficientDet, and the large version of YOLOv5. Each model’s learning rate was automatically defined by the Fastai^42^ learning rate finder. The Cascade R-CNN was implemented using Detectron2^43^. The meta learner models were applied using SkLearn and XGBoost library. The ensemble and stacking techniques use the results of the three best baseline models. All metrics were measured using Rafael Padilla’s tool^38^. In ensemble techniques, the Weighted Boxes Fusion tool was applied^36^.

The experiments were executed on a Linux system with an Intel Core i7-10750H CPU @ 2.60 GHz, a NVIDIA GeForce RTX 3080 Laptop GPU, and 16 GB of RAM.

## Results

The fivefold cross-validation is employed to evaluate each model’s performance, where the training set and the test set do not share the same images. As seen in Fig. 3, the StackBox algorithm, independently of the model used as meta-learner, achieves significantly higher results concerning mAP when compared to base learner models and ensemble techniques.

**Figure 5:**
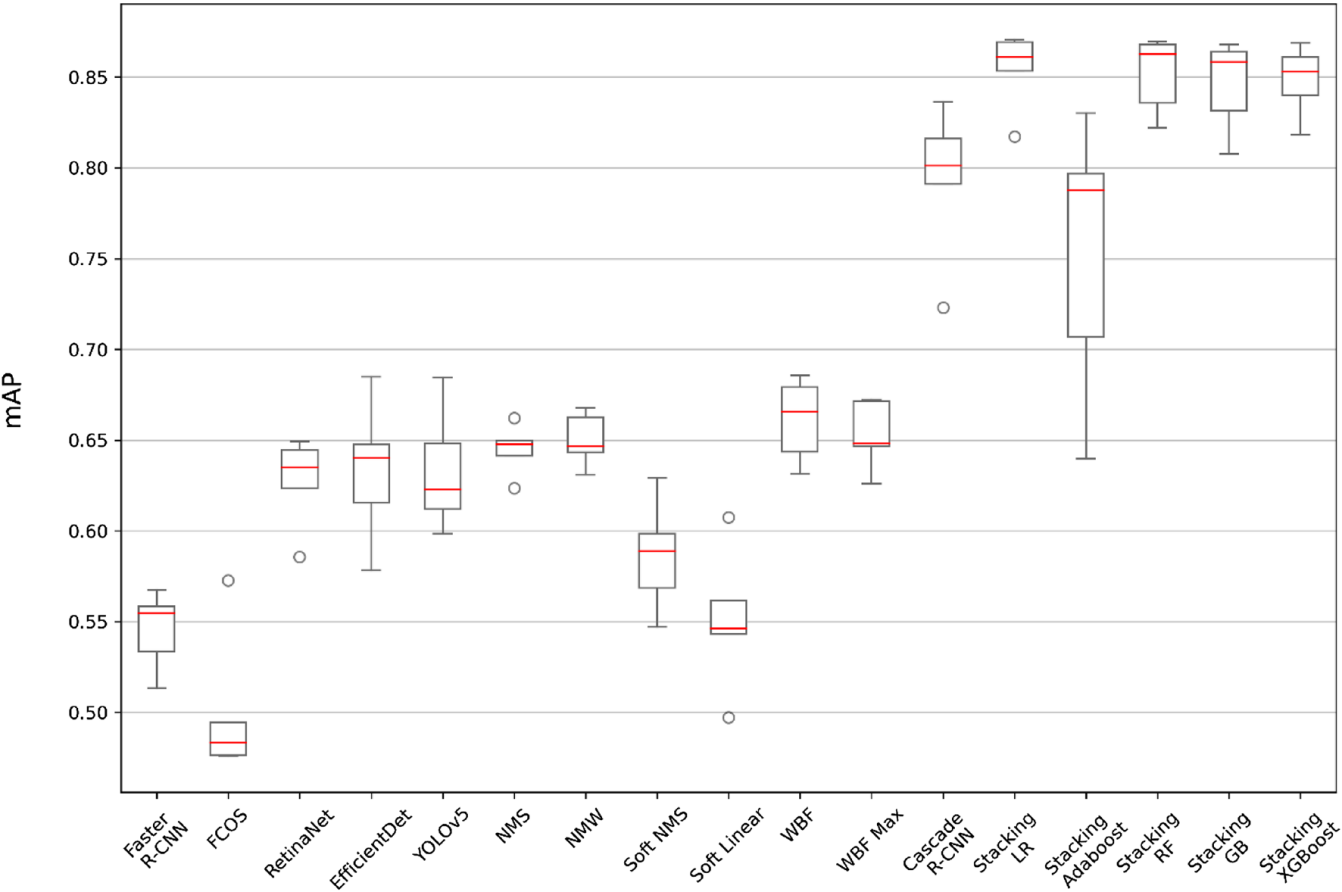
mAP comparison through boxplots. The tested models (base-learner models, ensemble models, Cascade R-CNN and StackBox models) are compared in terms of mAP. The figure shows the distribution of the results on the fivefold cross-validation.

Concerning mAP, EfficientDet, RetinaNet, and YOLOv5 achieve similar results, of around 0.63 on average. The WBF ensemble technique is able to improve this value to 0.66. Cascade R-CNN achieves an average mAP value of 0.79. Our proposal, StackBox, raises the mAP to 0.85 in all the meta-learners used, except for Adaboost, where the mAP is 0.75, as shown in Fig. 5.

Table 1 presents the results that average the five folds together concerning precision. In object detection, precision is a model’s capability to identify only relevant objects, corresponding to the percentage of correct positive predictions^38^.

**Table 1:** Model comparison in terms of precision.

Algorithm	AP@[.5:.05:.95]	AP@.50	AP@.75	AP_M_	AP_L_
Faster R-CNN	0.20 ± 0.01	0.54 ± 0.02	0.07 ± 0.01	0.02 ± 0.02	0.24 ± 0.02
FCOS	0.19 ± 0.01	0.50 ± 0.04	0.04 ± 0.01	0.08 ± 0.00	0.21 ± 0.01
RetinaNet	0.25 ± 0.01	0.63 ± 0.02	0.10 ± 0.01	0.03 ± 0.03	0.28 ± 0.02
EfficientDet	0.24 ± 0.02	0.63 ± 0.04	0.06 ± 0.02	0.03 ± 0.02	0.27 ± 0.03
YOLOv5	0.24 ± 0.02	0.63 ± 0.03	0.05 ± 0.03	0.02 ± 0.01	0.27 ± 0.02
NMS	0.24 ± 0.02	0.64 ± 0.02	0.06 ± 0.02	0.03 ± 0.03	0.27 ± 0.02
NMW	0.25 ± 0.02	0.65 ± 0.02	0.08 ± 0.02	0.03 ± 0.02	0.28 ± 0.02
Soft NMS	0.23 ± 0.02	0.58 ± 0.03	0.08 ± 0.01	0.03 ± 0.02	0.26 ± 0.03
Soft Linear	0.22 ± 0.02	0.55 ± 0.04	0.08 ± 0.02	0.03 ± 0.02	0.25 ± 0.03
WBF	0.26 ± 0.02	0.65 ± 0.02	0.09 ± 0.03	0.03 ± 0.01	0.29 ± 0.02
WBF Max	0.25 ± 0.02	0.65 ± 0.02	0.09 ± 0.03	0.03 ± 0.02	0.28 ± 0.03
Cascade R-CNN	0.64 ± 0.02	0.79 ± 0.04	0.72 ± 0.03	**0.31** ± 0.11	**0.69** ± 0.01
StackBox with LR	**0.65** ± 0.03	**0.85** ± 0.02	**0.75** ± 0.04	**0.31** ± 0.03	**0.69** ± 0.04
StackBox with Adaboost	0.35 ± 0.09	0.75 ± 0.08	0.29 ± 0.16	0.06 ± 0.03	0.39 ± 0.10
StackBox with RF	0.64 ± 0.03	0.85 ± 0.03	0.74 ± 0.04	**0.31** ± 0.02	**0.69** ± 0.04
StackBox with GB	0.62 ± 0.04	0.84 ± 0.03	0.72 ± 0.05	0.28 ± 0.03	0.67 ± 0.04
StackBox with XGBoost	0.63 ± 0.03	**0.85** ± 0.02	0.73 ± 0.04	0.29 ± 0.02	0.68 ± 0.04

The results present the average values obtained by combining the 5 folds ± SD of those results. AP@[.5:.05:.95] computes the average precision with 10 different IoU thresholds and takes the average among all computed results. In AP@.50 and AP@.75, the interpolation is performed in *N* = 101 recall points, and the first uses an IoU threshold equal to 0.5, whereas the second uses a threshold of 0.75. AP_M_ only evaluates medium-sized ground-truth objects, whereas AP_L_ only evaluates large ground-truth objects^38^. Bold denotes the highest values for each metric. The StackBox with Logistic Regression stands as the best model for all the metrics under consideration.

RetinaNet achieves the best results in terms of precision when comparing base learner models, but with a slight difference from EfficientDet and YOLOv5, as seen in Table 1. Faster R-CNN and FCOS achieve the worst performance. Considering ensemble techniques, we can see a subtle improvement for some of the techniques, with relevance to WBF, with an improvement of 0.02 in AP@[.5:.05:.95] and AP@.50 compared with the best base learner models. In our approach, independently of the meta learner algorithm used, except for Adaboost, we verify a significant improvement regarding the base learner models and the ensemble techniques. Cascade R-CNN achieves similar results to our StackBox technique in AP_M_ and AP_L_ but slightly worse results in the remaining measures. StackBox with LR increases precision to around 0.4 in AP@[.5:.05:.95] and in AP_L_, 0.7 in AP@.75, and 0.2 in AP@.50 and AP_M_ when compared to base learner models and the remaining ensemble techniques.

To compare the performance of all tested models concerning recall, we measure the performance of all models in various metrics usually applied in object detection research. Recall is the capability of a model to find all the ground-truth bounding boxes, corresponding to the percentage of correct positive predictions among all given ground truths^38^.

In Table 2, we can verify that results show similar results as precision. One clear difference is that Faster R-CNN achieves results similar to RetinaNet concerning the recall, whereas FCOS, is the worst model (e.g., in precision). Cascade R-CNN achieves similar results when compared with StackBox, but with lower performance in AR_10_ and AR_L_. StackBox with LR achieves the highest average values, with 0.65 in AR_1_, 0.71 in AR_10_, 0.34 in AR_M_, and 0.76 in AR_L_.

**Table 2:** Model comparison in terms of recall.

Algorithm	AR_1_	AR_10_	AR_M_	AR_L_
Faster R-CNN	0.30 ± 0.01	0.33 ± 0.01	0.07 ± 0.04	0.36 ± 0.02
FCOS	0.24 ± 0.02	0.24 ± 0.02	0.01 ± 0.01	0.27 ± 0.01
RetinaNet	0.31 ± 0.01	0.32 ± 0.01	0.06 ± 0.04	0.36 ± 0.02
EfficientDet	0.29 ± 0.01	0.30 ± 0.01	0.06 ± 0.02	0.34 ± 0.02
YOLOv5	0.29 ± 0.01	0.30 ± 0.01	0.08 ± 0.03	0.34 ± 0.01
NMS	0.31 ± 0.01	0.32 ± 0.01	0.08 ± 0.04	0.36 ± 0.02
NMW	0.32 ± 0.01	0.33 ± 0.01	0.08 ± 0.04	0.37 ± 0.01
Soft NMS	0.31 ± 0.01	0.35 ± 0.01	0.08 ± 0.04	0.39 ± 0.02
Soft Linear	0.31 ± 0.01	0.36 ± 0.01	0.08 ± 0.04	0.40 ± 0.02
WBF	0.31 ± 0.01	0.33 ± 0.01	0.08 ± 0.04	0.37 ± 0.01
WBF Max	0.32 ± 0.01	0.33 ± 0.01	0.08 ± 0.04	0.37 ± 0.01
Cascade R-CNN	**0.65** ± 0.02	0.69 ± 0.03	**0.34** ± 0.12	0.75 ± 0.02
StackBox with LR	**0.65** ± 0.03	**0.71** ± 0.03	**0.34** ± 0.03	**0.76** ± 0.04
StackBox with Adaboost	0.42 ± 0.08	0.44 ± 0.09	0.08 ± 0.04	0.49 ± 0.10
StackBox with RF	**0.65** ± 0.03	0.70 ± 0.03	**0.34** ± 0.02	0.75 ± 0.04
StackBox with GB	0.64 ± 0.03	0.69 ± 0.03	0.31 ± 0.03	0.74 ± 0.04
StackBox with XGBoost	**0.65** ± 0.03	0.70 ± 0.03	0.33 ± 0.03	0.75 ± 0.04

The results present the average values obtained by combining the 5 folds ± SD of those results. AR1 measures the average recall considering up to one detection per image, averaged over all IoUs, whereas AR_10_ considers 10 detections at most. Similar to precision, AR_M_ measures the average recall on medium-sized ground-truth objects, whereas AR_L_ only evaluates large ground-truth objects^38^. Bold denotes the highest values for each metric. The StackBox with Logistic Regression stands as the best model for all the metrics under consideration.

## Discussion

Many studies have demonstrated the suitability of object detection approaches for efficiently detecting polyps. Different algorithms have been tested, and to achieve better results on the task, ensemble techniques combining the predictions of these algorithms have been proposed. Knowing that different algorithms have their specificities, advantages, and disadvantages, the results can significantly differ when considering the precision, recall, and mAP of the resulting models. Following this reasoning, in this study, we demonstrate that the stack of predictions from separate object detection algorithms improved the precision of polyp detections. Independently of the meta learner used, the mAP increased significantly compared to base learner algorithms such as EfficientDet and RetinaNet, prior ensemble techniques such as NMS and WBF, and multistage architecture Cascade R-CNN.

To the best of our knowledge, this is the first stacking approach to combine the predictions of the coordinates of different object detection algorithms. In the context of this study, the technique was applied to polyp detection. However, it can be easily used in other medical applications and, in general, in all the problems in which the precision of the localization of objects of interest is the main concern.

Due to the different natures of the algorithms used, the predictions of each base model are computed differently, leading to different bounding boxes. We can use this dissimilarity and the advantages of each algorithm to combine them in a more precise prediction.

Regarding the mAP, the base learner with the highest value is RetinaNet, with an average mAP of 0.63, whereas the WBF ensemble technique can increase this value to an average of 0.66 and Cascade R-CNN can improve this value to 0.79. Our proposal, StackBox with LR, achieves an average mAP of 0.85, representing an increase of 0.22 compared to RetinaNet, 0.19 compared to WBF, and 0.06 compared to Cascade R-CNN.

Concerning precision, EfficientDet, RetinaNet, and YOLOv5 are the three best base learner models for most of the considered metrics. Using ensemble techniques, we can improve those results by around 0.02, and, with Cascade R-CNN, we achieve slightly worse results when compared to StackBox. Our approach can increase the precision of the models significantly. Considering stacking with LR, we double the performance (for most metrics) compared to base learner models.

Concerning recall, FCOS presents the worst results compared to the other baseline models. Faster R-CNN, RetinaNet, EfficientDet, and YOLOv5 achieve similar results, with approximately 0.3 in AR_1_ and AR_10_, 0.07 in AR_M_, and 0.35 in AR_L_. Prior ensemble techniques can slightly improve those values, but StackBox increases AR_1_ to 0.65, AR_10_ to 0.71, AR_M_ to 0.34, and AR_L_ to 0.76. Cascade R-CNN presents slightly worse results than StackBox does.

**Figure 6:**
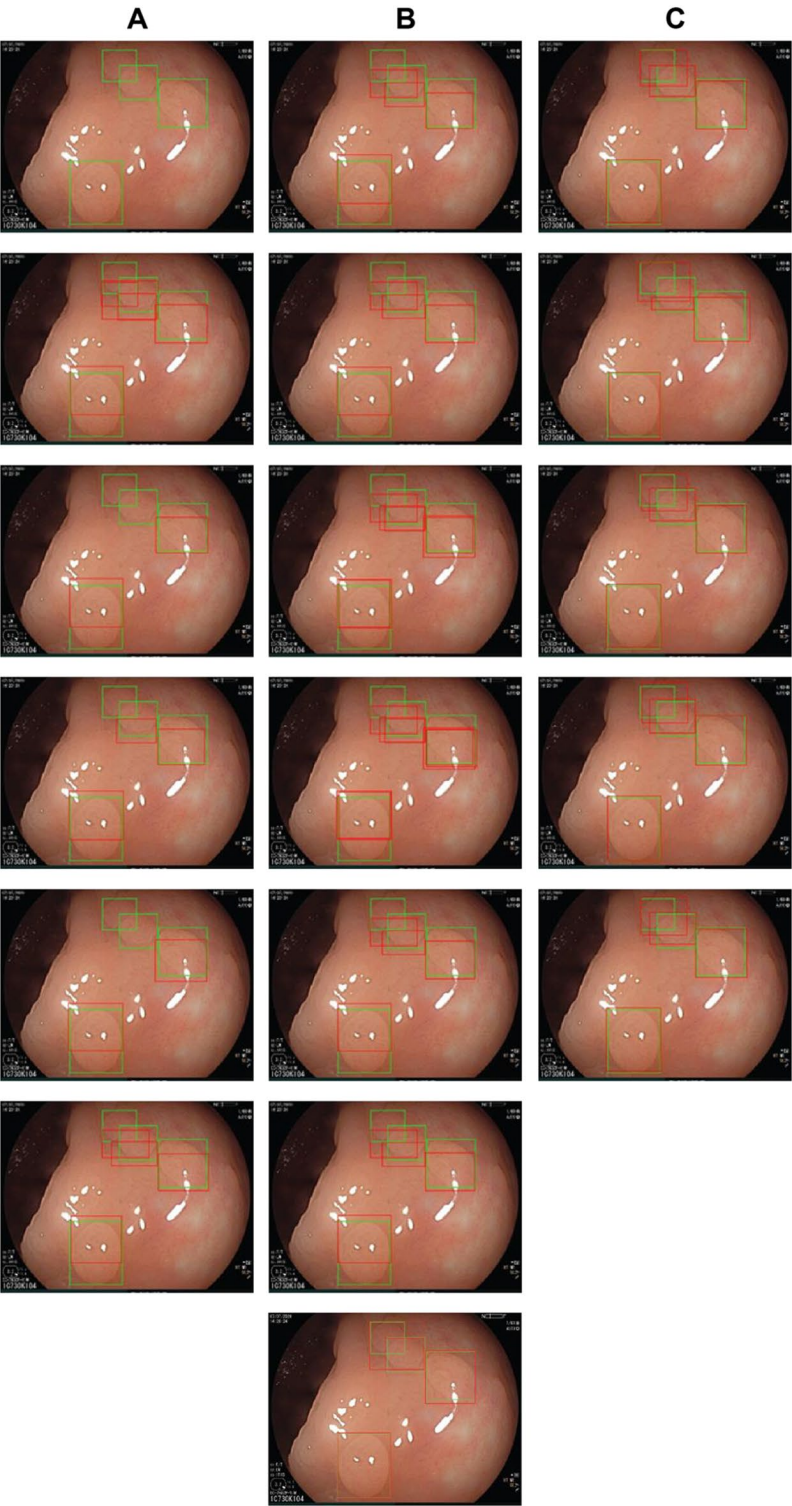
Predictions comparison sample. Column A displays, from the top to the bottom, the results of the following models: GT, Faster R-CNN, FCOS, RetinaNet, EfficientDet, and YOLOv5. Column B evaluates the predictions using ensemble techniques (NMS, NMW, SOFT-NMS, Soft Linear, WBF, and WBF Max) and Cascade R-CNN. The third column reports the results of our stacking technique using different meta-learner models: StackBox with Logistic Regression, StackBox with Adaboost, StackBox with Random Forest, StackBox with Gradient Boosting, and StackBox with XGBoost. The ground truth is represented by the green bounding boxes and the predictions by the red bounding boxes.

Figure 6 shows the results achieved, on a sample image, by the models considered in this study. Clearly, StackBox, independently of the meta learner used, stands as the best performer, with significant improvement in the precision of the predicted boxes compared to the other methods under consideration.

Regarding the real-time applicability of this approach and to validate the practical usefulness of StackBox in real-world colonoscopy, we evaluate the processing time for each image. When we apply StackBox, the inference on new images includes the inference of each base learner model in the new data, the manipulation of those predictions in a format viable to apply stacking techniques, the stacking technique itself, and finally, the implementation of a NMS strategy to remove redundant boxes. For the example given, where we use EfficientDet, RetinaNet, and YOLOv5, the inference time is approximately 0.054, 0.057, and 0.010 s per image, respectively. The prediction manipulation to obtain the needed format for stacking application requires around 0.010 s per image. The inference during the stacking approach when implementing a LR demands 0.00048 s per image, and the NMS application requires around 0.020 s per image. Summing up all the procedures needed to obtain the final predictions, we obtain an inference time of 0.144 s per image, translating into around seven frames per second. This value is considered lower than inference times associated with widely used algorithms, such as the Faster R-CNN Inception ResNet V2 640 × 640 (0.206 s/image)^44^.

This study poses the basis for further solutions to this challenging problem. In future works, this methodology can be applied to data sets with a larger number of samples (to improve the performance of the base learners), and more advanced strategies to combine the predictions of the base learners can be defined and analysed.

## Conclusion

To achieve better results on the polyp detection task, in this paper, we proposed the use of StackBox. StackBox combines the predictions on training data sets from YOLOv5, RetinaNet, and EfficientDet by stacking the results with a meta-learner, aiming to build a model that can increase the detection capability over new data. Experimental results demonstrated the suitability of the proposed method for the polyp detection task. More specifically, StackBox can significantly improve the mAP of the detections, not only when compared to the tested baseline models, namely Faster R-CNN, FCOS, YOLOv5, RetinaNet, and EfficientDet, but also with respect to existing ensemble techniques, namely NMS, Soft-NMS, NMW, and WBF, and the multistage architecture Cascade R-CNN. These results, obtained by considering distinct metrics commonly used in object detection problems, demonstrate that StackBox is superior to all the tested approaches.

We believe that the proposed algorithm may contribute to successful colonoscopy procedures by reducing the polyp miss rate due to the increase in detection precision; furthermore, by combining several object detection frameworks with different skills on the task, we obtain different predictions, which will provide a more robust model with a higher polyp detection capability. Thus, StackBox can be considered a procedure of significant relevance to CRC prevention using deep learning techniques, and the feasibility of the approach in real-world clinical practice is supported by its short inference time on new data.

The results achieved in this study open a wide range of future research directions, including the construction of generalizable models to deal with various object detection tasks.

## Data Availability

The datasets generated during and/or analysed during the current study are available in the BK.AI repository, https://bkai.ai/research/bkai-igh-neopolyp-small-a-dataset-for-fine-grained-polyp-segmentation and in https://www.kaggle.com/c/bkai-igh-neopolyp.
